# Medical Evidence Influence on Inpatients and Nurses Pain Ratings Agreement

**DOI:** 10.1155/2016/9267536

**Published:** 2016-04-28

**Authors:** Boaz Gedaliahu Samolsky Dekel, Alberto Gori, Alessio Vasarri, Maria Cristina Sorella, Gianfranco Di Nino, Rita Maria Melotti

**Affiliations:** ^1^University of Bologna, Department of Medicine and Surgery Sciences, Via Massarenti 9, 40138 Bologna, Italy; ^2^Azienda Ospedaliera-Universitaria di Bologna Policlinico S. Orsola-Malpighi, Via Massarenti 9, 40138 Bologna, Italy; ^3^University of Bologna, Post-Graduate School of Anaesthesia and Intensive Care, Via Massarenti 9, 40138 Bologna, Italy

## Abstract

Biased pain evaluation due to automated heuristics driven by symptom uncertainty may undermine pain treatment; medical evidence moderators are thought to play a role in such circumstances. We explored, in this cross-sectional survey, the effect of such moderators (e.g., nurse awareness of patients' pain experience and treatment) on the agreement between *n* = 862 inpatients' self-reported pain and *n* = 115 nurses' pain ratings using a numerical rating scale. We assessed the mean of absolute difference, agreement (*κ*-statistics), and correlation (Spearman rank) of inpatients and nurses' pain ratings and analyzed congruence categories' (CCs: underestimation, congruence, and overestimation) proportions and dependence upon pain categories for each medical evidence moderator (*χ* ^2^ analysis). Pain ratings agreement and correlation were limited; the CCs proportions were further modulated by the studied moderators. Medical evidence promoted in nurses overestimation of low and underestimation of high inpatients' self-reported pain. Knowledge of the negative influence of automated heuristics driven by symptoms uncertainty and medical-evidence moderators on pain evaluation may render pain assessment more accurate.

## 1. Introduction

Pain evaluation is fundamental for appropriate management. Nonetheless, caregivers are often inaccurate in their pain assessments and tend to misjudge pain [1, 2]. While patient self-reported pain (PSRP) appears to provide the most valid pain measure, observer-rated pain is often biased and incongruent with PSRP [2, 3]. Congruence studies which compare observer-rated pain with PRSP confirm that caregivers frequently misestimate patient pain and that incongruence may include both under- and overestimation of PSRP [3–6]. Misestimation may depend upon inaccurate evaluation, biased assessment, or both. While inaccuracy often relates to the caregiver skills and assessment tool reliability, assessment may be biased by patient, caregiver, and situational moderators [5, 7–12].

The study of the relationships between the moderators that are thought to bias pain assessment and the features of the congruence between PSRP and observer-rated pain can be used as a tool to uncover the impact of pain assessment moderators on pain estimation and possibly to offer clues for the explanation of pain misestimation [2, 4]. Congruence features include the mean of the absolute difference, proportions of congruence categories (CCs: underestimation, congruence, and overestimation), the amount of agreement, and the correlation between PSRP and observer-rated pain [3, 4, 13]. Evidence shows that patient and setting moderators influence the congruence between PSRP and nurse evaluation of a patient's pain (NEP) [3, 14]. Previously we have found PSRP-NEP congruence to be limited, to include both under- and overestimation of PSRP, and to be further influenced by PSRP intensity and psychosocial (age, gender, and marital status) and situational (admission area and hospital stay) moderators [3].

Among situational moderators the medical evidence, relevant to the patient's condition and available to the caregiver when making symptom judgments, was proposed as a possible bias source for PSRP-NEP congruence. Medical evidence includes all medical findings: symptoms, diagnosis, and treatments. Evidence supports its influence on symptom judgments made by lay and medical observers and in different clinical and research contexts [3–5, 12, 15, 16]. In its presence, observers' ratings of chronic pain were reported to be much higher than in its absence; its absence was salient to observers primarily for high PSRP. Previously we have found that, among inpatients, pain assessment was influenced by both medical and pain treatment evidences: in the presence of robust medical evidence for severe pathology, nurses inflated low PSRP, but because these patients received pain treatment, nurses strongly discounted high PSRP; similarly, clinical and treatment evidences were found to inflate overestimation of low PSRP in oncology, surgery, and ICU wards [3]. Thus, the caregiver's awareness of the patient pain experience and treatment may represent a cognitive bias source in pain evaluation especially in the presence of symptom uncertainty. Indeed, when symptom certainty is low (i.e., the PSRP validity is, for any reason, questionable), clinical judgments are more ambiguous and patients may be vulnerable to pain misestimation as observers may incorrectly use pain cues (e.g., medical evidence) and heuristics that may simplify clinical decisions but will bias pain estimation and treatment [4, 17]. As such cognitive bias can lead to systematic deviations from a standard of rationality or good judgment in pain evaluation and thus it is important to uncover their nature and source.

This study was set to uncover, in our hospital setting, possible relations between some selected medical evidence moderators and PRSP-NEP congruence features in order to assess the hypothesis that the exposure to such moderators may bias nurses against reporting pain in others. In particular, we sought to explore the potential affect of variables related to nurses' awareness of patients' pain experience and treatment in the past 24 h on PRSP-NEP congruence features. As recommended [4], results may add knowledge and explanation to the role of such variables as predictors of bias in congruence studies and in pain evaluation.

## 2. Methods

### 2.1. Settings, Participants, and Procedures

All inpatient hospital wards (except neonatology, paediatric intensive care, emergency room, and psychiatry), *n* = 862 inpatients and *n* = 115 nurses were included in the study.

Two days before Index day, the chief nurse of each ward appointed and instructed the nurses who were to participate in the study as pain assessors. These nurses had to meet two conditions: to be actively operating in the ward for at least 7 days and to have worked one shift in the 24 h prior to the Index day. Each assessor was appointed to assess no more than 10 patients. As the study started, chief nurses established the list of the eligible patients. Inclusion criteria were patients ≥ 6 years of age, hospitalization for at least 24 h at the time of the study, giving consent, and having no cognitive impairments or language/speech barriers. Thereafter, they administered the questionnaire to these patients for pain self-estimation. Simultaneously, assessors reviewed these patients' charts for demographic and medical data; then, without directly questioning the patients, they estimated these patients' current pain. All pain ratings were made using a 0 (“no-pain”)–10 (“worst pain I can imagine”) numerical rating scale (NRS); nurse estimation of pain will be referred to as NEP while patient self-reported pain will be referred to as PSRP. Assessors made their assessment by using the information acquired both during their professional interaction with the patient prior to the study and from the medical chart. This information included the moderators whose relations with PSRP-NEP congruence features were to be analysed.

### 2.2. Analysis Rationale and Data Management

To be meaningful, congruence studies should include the Pearson correlation, the proportions of congruent scores, and the mean of the absolute difference (MAD) between patient and observer [13]; Iafrati suggested that patient/observer VAS ratings may be considered concordant if they differ by less than 1 cm [18]. The use of correlation and regression alone is inappropriate to describe agreement between two observers who measure the same variable as there might be a close correlation but poor clinical agreement [3, 4]. Moreover, for ordinal data (like the NRS), transformation in categories and a nonparametric correlation method, such as the Spearman rank, should be used and, for category variables, agreement is determinable by the *κ*-statistics [19]. The Iafrati criterion applicability was questioned [3, 4] and thus it was not used in this study. Indeed, it may induce artificial agreement between pain categories; as either category implies different clinical approaches, confusion between them may induce treatment and epidemiological errors. In our study NRS scores were first used to determine PSRP-NEP MAD and, hence, transformed into pain categories to enable computation of Spearman rank correlation, *κ*-statistics, and congruence categories proportions.

The primary goals of the study were to assess the following NEP-PSRP congruence features: MAD (on a 0–10 scale), agreement, correlation, and proportions of congruence categories (CCs: underestimation, congruence, and overestimation). The secondary goal was to analyse these features with relation to independent category variables, namely, “congruence moderators” (see below). For these purposes, NRS scores of both NEP and PSRP were transformed into four pain categories: no-pain (NRS = 0), mild pain (NRS ≥ 1 and ≤3), moderate pain (NRS ≥ 4 and ≤6), and severe pain (NRS ≥ 7), following Collins et al. [20]. Finally, for each patient, NEP and PSRP categories were compared and proportions of congruence (NEP = PSRP), underestimation (NEP < PSRP), or overestimation (NEP > PSRP) were calculated. Establishing the aforementioned congruence features enabled the evaluation of their relationship with independent variable categories like the PSRP categories and the “congruence moderators” described below.

### 2.3. Congruence Moderators

Aside PSRP, congruence moderators' information was stated by the assessors and hence may not necessarily coincide with the patient opinion. Moderators included

1. PSRP categories (*no-pain, mild, moderate*, and* severe* pain);
2. patients' pain experience moderators: pain in the past 24 h (yes/no); cause of pain (surgery, trauma, diagnostic procedures, cancer, other, and unknown);
3. pain treatment moderators: analgesics prescription in the past 24 h (yes/no); analgesics administration schedule: around the clock (*ATC*), on demand (*PRN*), and* ATC* +* PRN*; analgesic type: nonsteroidal anti-inflammatory drugs (NSAIDs), opioids, and NSAIDs + opioids (yes/no).

### 2.4. Ethics

The study was authorized by the Hospital Direction, approved by its Ethics Committee and conducted according to the Declaration of Helsinki and IASP's guidelines for pain research in animals and humans. All participants were personally and thoroughly informed by the investigators on the aims and the structure of the survey. Patients and nurses were informed that participation in the survey was voluntary and anonymous and would not affect their ongoing therapy and work, respectively. An informed consent was obtained from adult patients and from parents or legal guardians for patients <18 years.

### 2.5. Data Presentation and Statistical Analysis

Continuous data were reported as the mean (±standard deviation). MAD was reported as the mean and (95% CI, upper and lower confidence intervals) category data and proportions were expressed in percentages. The CCs dependence upon independent variable categories (e.g., PSRP categories) was determined using *χ* ^2^-analysis. When significant, a* post hoc* cell contribution analysis was performed and major contributions for the association were reported. The analysis of agreement and correlation between NEP and PSRP categories were performed by the *κ*-statistics and the Spearman rank correlation methods, respectively. When statistically significant, an absolute *κ* value between 0.1 and 0.3 was considered as mild agreement; 0.31–0.5 as moderate; and 0.51–1.0 as excellent [19]. For the correlation analysis, when statistically significant, an absolute Rho (*ρ*) value between 0.2 and 0.4 was considered as mild association; 0.41–0.7 as moderate; and 0.71–1.0 as strong [19]. Statistical significance was defined as *P* < 0.05. When appropriate, *P* < 0.01 and *P* < 0.001 were reported.

## 3. Results

PSRP was obtained from *n* = 862 patients and *n* = 115 nurses provided the NEP for these patients; mean assessor/patients ratio was 1 : 7.5. Eighteen eligible patients were not enrolled (*n* = 11 were out of the ward during the study and *n* = 7 refused participation). Patients' mean age was 61.3 (±20.9) years and 52.2% (*n* = 450) were females.  Table 1 describes, for each congruence moderator subset, the MAD between NEP and PSRP NRS scores, the proportions of NEP-PSRP pain categories agreement and disagreement (under- and over-estimation), and the results of the *κ*-statistics and Spearman correlation analyses.  Table 2 reports the distribution and proportions of NEP and PSRP pain categories in the sample. PSRP and NEP category proportions and distribution in the sample were, respectively,* no-pain*, 62.0/50.3% (*n* = 535/434);* mild*, 12.1/23.1% (*n* = 104/199);* moderate*, 15.0/17.9% (*n* = 129/154), and* severe*, 10.9/8.7% (*n* = 94/75).

### 3.1. Awareness for Patients' Pain Experience

According to the assessors 53.5% of the patients experienced pain in the past 24 h. Agreement within this subset was half of that found within the subset of patients who, according to the nurses, were without pain experience (Table 1). In both subsets NEP-PSRP correlation was moderate while agreement was only mild or poor, respectively.

CCs dependence upon the presence or the absence of pain experience was statistically significant (*χ* ^2^ = 125.217, d.f. = 2, and *P* < 0.001); major contributions for this dependence were the association of* overestimation* with the presence of pain experience and the association of* congruence* with the absence of pain experience.

### 3.2. Causes of Pain

According to the assessors, the most common causes of pain were surgery (37.5%) and those which were not specified in the questionnaire (other, 36.2%). These were the only subsets in which agreement was statistically significant although it was poor or mild, respectively (Table 1).

NEP-PSRP correlation was found to be mild within the* surgery* subset and moderate within the* diagnostic procedures* and the* other* subsets.

### 3.3. Analgesics Prescription

Out of the *n* = 855 patients for whom nurses have indicated the presence/absence of analgesics prescription only 21% had such prescription. NEP-PSRP agreement and correlation in both subsets were mild and moderate, respectively (Table 1). CCs dependence upon both subsets was statistically significant (*χ* ^2^ = 40.153, d.f. = 2, and *P* < 0.001); major contribution for this association was the association of* congruence* with the absence of analgesic prescription followed by that of* overestimation* with the presence of such prescription.

### 3.4. Analgesics Administration Timing

Analgesic administration scheme was known for *n* = 173 patients. Administration schemes were* ATC* (37.0%),* PRN* (54.9%), and the association of both* ATC* and* PRN* (8.1%). Agreement, though limited, was statistically significant only for the* ATC* regimen while correlation was found to be mild and moderate for the* PRN* and *ATC* + *PRN* regimens, respectively (Table 1).

CCs were found to be independent of the administration regimen. Yet, proportions of* overestimation* were the highest (50%) for the* PRN* with or without* ATC* regimen (Table 1). It was noted that underestimation proportion was the lowest for the PRN + ATC regimen.

### 3.5. Analgesic Type

Among the *n* = 179 patients with analgesic prescription 57.5% had NSAIDs prescription, 35.2% had opioids, and 7.3% had both. In general, CCs were shown to be independent of the analgesic drug prescribed (*χ* ^2^ = 3.222, d.f. = 4, and *P* > 0.05). Nonetheless, qualitative differences in CCs proportions in each drug subset could be detected. As reported in Table 1,* overestimation* was shown in almost half of the patients with an NSAIDs or opioids prescription whereas* congruence* was found in almost half of the patients with an NSAIDs + opioids prescription. Proportions of* overestimation* in the latter subset were half of those found in either the NSAIDs or opioids prescription subsets.

As shown in Table 1, only NSAIDs prescription, whether combined with opioids or not, promoted statistically significant NEP-PSRP agreement and correlation. Interestingly, in the latter case NEP-PSRP correlation was found to be strong.

### 3.6. CCs Dependence upon PSRP Categories

Table 3 reports, for each moderator subset, the results of the *χ* ^2^-analysis for CCs dependence upon PSRP categories and the major contribution for such dependence (*post hoc* analysis). The* mild* and* moderate* PSRP categories are not shown in this table as CCs/PSRP associations were found only for* no-pain* and* severe* PSRP categories. In 13.5% of the moderator subsets CCs were either found to be independent from the PSRP categories or there was no sufficient data to complete this analysis. In the majority of the subsets in which CCs were found to be dependent upon the PSRP category,* underestimation* was associated with the* severe* PSRP category; the two exceptions were the association of* congruence* and* no-pain* PSRP category for the “absence of pain experience in the past 24 h” subset and the association of* overestimation* and* no-pain* PSRP category for the subset “others” among “cause of pain” moderator.

**Figure 1:**
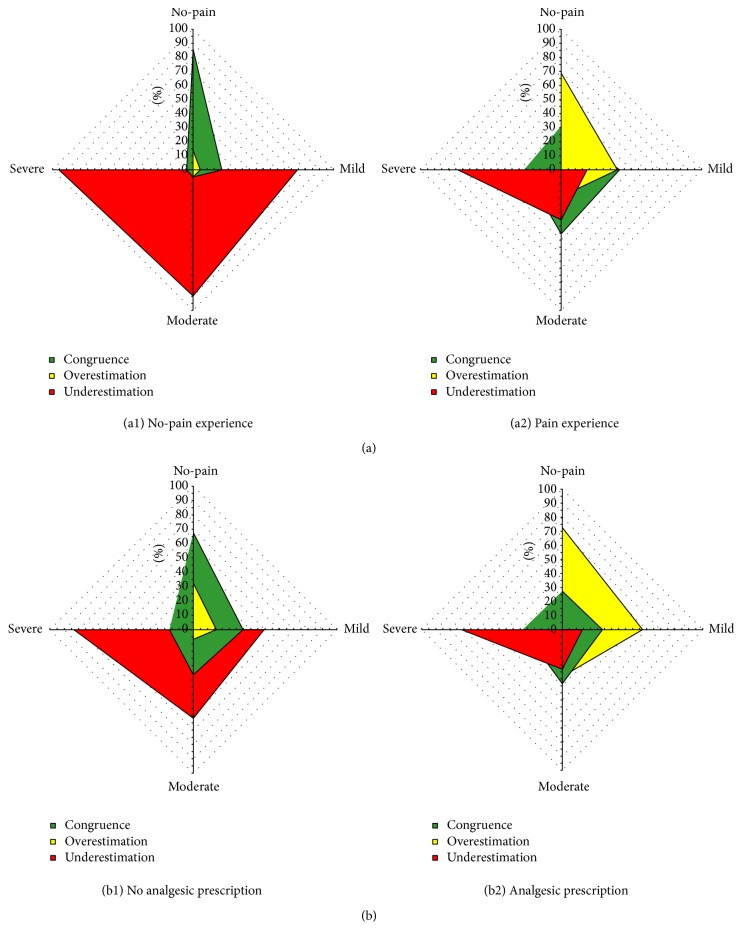
Radar plots of CCs proportions in each PSRP category within the subsets of patients with and without pain experience (a) and within the subsets of patients with and without analgesic prescription (b).

For more detailed qualitative observation, Figure 1 shows radar plots of the CCs proportions in each PSRP category within the subsets of patients that were, according to the assessor, with or without pain experience in the past 24 h ((a), (a2) and (a1), resp.) and within the subsets of patients with or without analgesics prescription ((b), (b2) and (b1), resp.).

In particular, while for both “pain experience in the past 24 h” subsets CCs were variably yet significantly associated with PSRP categories (Table 3), within the subset of patients without pain experience (versus the opposite subset), the proportions of congruence for* no-pain* category and of* underestimation* for mild to* severe* PSRP categories were particularly high [74% and 96%, resp.; see Figure 1 ((a), (a2) and (a1))].

Interestingly, major differences between the subsets of the presence or absence of analgesics prescription were detected when CCs proportions within each PSRP category were compared. Indeed, while for patients with analgesic prescription* congruence* proportions in all PSRP categories were low and roughly similar (Figure 1(b2)), this was not the case for patients without analgesic prescription (Figure 1(b1)). In the latter subset the* congruence* proportions within the* no-pain* PSRP category were more than twofold higher and those of* underestimation* of* mild* and* moderate* PSRP categories were more than two- and threefold higher, respectively. Thus,* congruence* with the* no-pain* PSRP category and* underestimation* of* mild* and* moderate* PSRP categories were prominent features in the absence of analgesic prescription. On the contrary, for patients with analgesic prescription (Figure 1(b2)),* overestimation* was the most representative feature. Indeed its proportions within all PSRP categories were higher than in those of patients without analgesic prescription.

Finally, CCs dependence upon the PSRP category was statistically significant in both the* ATC* and* PRN* subsets but not within the *PRN* + *ATC* subset. Major contributions for this dependence were the associations of* underestimation* and* severe* pain category followed by that of* overestimation* and* no-pain* category.

## 4. Discussion 

While most congruence studies are restricted to predictable pain settings [12, 15, 16, 21–29] or use vignette investigations with imaginary circumstances [23, 28, 30, 31], this study was held in an authentic comprehensive clinical context. It supports the hypothesis that situational features, like medical evidence moderators, relate to PSRP-NEP CCs and extends it to the entire inpatient population. We previously found that among inpatients PSRP-NEP agreement was limited; underestimation was directly proportional to the PSRP severity while congruence and overestimation were inversely proportional to it [3]. In this study we analysed the PSRP-NEP agreement profile, CCs proportions, and CCs dependence upon PSRP categories as a function of nurses' awareness of patients' pain experience and treatment in the past 24 h. Our results add evidence to the notion that these moderators may represent a source of bias during pain evaluation and offer elements for the explanation of pain misestimation. It is plausible, especially under conditions of symptoms uncertainty and ambiguous clinical judgment, that observers may incorrectly use medical evidence cues and follow established cognitive pathways to intuitive heuristics that may simplify clinical decisions but will bias pain estimation. Understanding of the negative influence of such heuristics on pain evaluation and avoiding them may render pain assessment more accurate.

Nurses' awareness of patients' pain experience in the past 24 h (versus its absence) showed a better agreement profile. However, it yielded higher proportions of pain overestimation (44%) and was associated with* underestimation* of* severe* pain. The assessor's assertion of the absence of pain experience promoted more robust proportions of PRSP-NEP* congruence* (64%) which were associated with* no-pain* PSRP category. Either* absence* or* presence* of analgesic prescription in the past 24 h yielded similar agreement profiles;* absence*, however, showed higher proportions of* congruence* (55%) while the* presence* of analgesic prescription showed higher proportions of pain* overestimation* (46%). Both subsets were associated with* underestimation* of* severe* pain. Among pain causes the subset “*other*” (i.e., causes not included among common causes list) showed the best, yet limited, agreement profile, the highest proportions of pain* underestimation* (42%), and association with* overestimation* of* no-pain* category. Both surgery and diagnostic procedures subsets yielded higher proportions (50%) of PRSP-NEP* overestimation* and* congruence*, respectively, and were associated with* underestimation* of* severe* pain.

These results seem to depict a trend for which both the presumed presence of pain experience and the analgesic prescription promoted low PSRP* overestimation* while their absence promoted PSRP-NEP* congruence*. In either case,* underestimation* of* severe* pain continued to be a prominent feature. These findings are consistent with other studies in which, for low, intermediate, or high PSRP, observer pain ratings were generally higher, equal, and lower, respectively [3, 5, 12, 17, 22, 23, 32, 33]. Clinically, overestimation is as harmful as underestimation: patients who report high PSRP even when nurses are aware of their pain experience and analgesic treatment are vulnerable to underestimation and hence to pain undertreatment [17]. Patients who report low PSRP are subject to overestimation and thus are exposed to overtreatment with potential treatment hazards.

One particular aspect faced in this study was the influence of pain treatment details (analgesic class and administration regimen) on PSRP-NEP agreement. We included this analysis to verify whether nurses' awareness of such moderators may further influence PSRP-NEP agreement. We sought to verify whether progressively stronger analgesics (NSAIDs versus opioids versus opioids + NSAIDs) and a more complex administration regimen (*PRN* versus* ATC* versus* ATC* +* PRN*) may represent medical evidence cues of patient pain for nurses and thus influence their evaluations.

Agreement profile in most pain treatment subsets was limited and for some subsets agreement and correlation were not statistically significant. Exceptions with fair agreement profiles were the prescription of opioids + NSAIDs with complex administration regimen (*ATC* + *PRN*). Taken separately any of NSAIDs, opioids, or complex administration regimen (*ATC* + *PRN*) subsets yielded high proportions of pain overestimation. Interestingly, a complex opioid administration regimen (*ATC* + *PRN*) yielded high proportions of underestimation. The highest proportion of PSRP-NEP agreement (67%) was found for the* ATC* opioids + NSAIDs prescription; this subset however included only 3 cases.

These results, although complex, seem to depict a comprehensible trend. Taken separately, the stronger the analgesic was or the more complex the administration regimen was, the higher the pain overestimation was. Put together, the stronger the analgesic was associated with more complex administration regimen, the higher the pain underestimation was. Analgesic associations and low-complex administration regimen promoted PSRP-NEP agreement. Explanation for these results may be that nurses may infer cues on pain severity from its treatment details and use them when making symptom judgments. Nonetheless, these cues may promote a dichotomous interpretation in the assessors: weaker analgesics (NSAIDs) and low complexity of their administration regimen may be interpreted as a confirmation for the underlying pain condition; stronger analgesics (opioids) and high complexity of administration regimen may confirm the underlying pain condition but, given the “vigorous” treatment, high PSRP severity may be questioned by the assessor. Thus, as the presence of any type of pain treatment was probably used by the nurses as a cue for the very presence of the patient's pain condition, nurses tended to inflate low PSRPs; however, if the patients received complex pain treatment, nurses strongly discounted high PSRPs.

Pain assessment can be demanding especially in patients who are unable to self-report. In such situations, relying on observational assessment tools is an alternative strategy [34]. The evidence affirms that as pain cannot be proved or disproved, PSRP should be accepted as the most reliable evidence for the pain presence and intensity [35, 36]. Often however, even when PSRP is available, symptom certainty may be undermined by a variety of reasons: PSRP may be incompatible with objective diagnostic findings and the subjective nature of pain assessment as well as medical treatments and standards of care may be inefficacious. To reduce uncertainty that often affects pain assessment, caregivers tend to follow established cognitive pathways to intuitive heuristics that serve to simplify the clinical judgments that they make; yet, those heuristics are also responsible for bias in symptom judgment especially when those judgments regard the certainty that caregivers attach to PSRP, and the information engaged to drive such heuristics frequently has little association with pain severity [17].

Evidence supports the influence of medical evidence on symptom judgments made by both lay and medical assessors [3, 12, 15–17, 27]. For example, among the inpatient population, the highest proportions of agreement were found in obstetrics and the lowest in surgery, oncology, and radiotherapy wards [3]. In these areas complex pain conditions and treatments are common. For obstetrics, high PSRP joined with the medical evidence of parturition (i.e., nurses' awareness of patients' pain experience) yielded the highest congruence proportions and low underestimation. For radiotherapy, high PSRP joined with the patients' serious health conditions did not promote congruence; underestimation was the highest while overestimation was significantly associated with low PSRP. Again it appears as if the medical evidence association with CCs was mitigated for high PSRP and amplified for low PSRP. As patients in radiotherapy had robust medical evidence for severe pathology, nurses inflated low PSRPs, but because these patients received robust pain treatment (i.e., nurses' awareness of pain treatment), nurses strongly discounted high PSRPs.

It was argued that, in symptoms judgment, medical evidence power may reside in its intuitive appeal mainly when judgment is subject, for any reason, to uncertainty. It provides a strategy, for symptoms judgment, that has the weight and reliability of “medicine” behind it, as codified in medical guides [37]. Nonetheless, as our results show, such behaviour induces bias in pain evaluation, undermines PSRP-NEP agreement, and may promote both under- and overestimation of PSRP and thus may lead to inappropriate treatment.

## 5. Study Limitations and Conclusions

As this study focused on PSRP-NEP agreement and correlation, CCs proportions, and relationships among variables, cause and effect conclusions could not be drawn. Further, these relationships might have been mediated by other moderators which were not addressed in this study (e.g., nurse's psychosocial and professional features and patient's verbal and nonverbal pain behaviours). The stratification of cases in some of the moderator subsets was limited by the number of the inpatients available. Further, the number of univariate analyses made in this study may increase the risk of Type I error. To allow interpretation, the level of *P* value, for each analysis, was reported. As many of these values were of high significance this risk is low. The study's ecological and external validity may be questioned. Indeed, the study design of indirect NEP modifies the direct evaluation method. Evidence shows that the availability of the PSRP to assessors does not guarantee PSRP-NEP agreement. If the study had focused on the nurses' judgment of the PSRP then the direct evaluation would have been essential to the nurse and its absence would have affected the ecological validity of the study task [3]. Data collection in this study was within a setting of routine work. Thus, the external validity of the study comes from its strong relevance to practice and the ability to highlight important assessment issues.

In this study of the entire inpatient population, the PSRP-NEP agreement was limited. CCs were further associated with situational moderators like nurses' awareness of patients pain experience and treatment. An understanding of the negative influence of automated heuristics driven by symptoms uncertainty and medical evidence moderators on pain evaluation and avoiding them may render pain assessment more accurate.

## Figures and Tables

**Table 1:** Summary of NEP-PSRP mean of NRS absolute differences, pain categories agreement/disagreement, and correlation.

Moderators^f^		NRS	Pain categories						
		Absolute differences	Agreement/disagreement (%)			*κ*-statistics		Spearman correlation coefficient	
		[mean (95% CI)]	Underestimation	Agreement	Overestimation	*κ*	Agreement based on *κ*	*ρ*	Correlation based on *ρ*
Pain experience in the past 24 h									
No	401	1.3 (1.1–1.5)	21.2	67.3	11.5	0.08^b^	Poor	0.55^a^	Moderate
Yes	461	2.6 (2.4–2.8)	21.0	34.7	44.3	0.14^a^	Mild	0.40^a^	Moderate
Cause of pain									
Surgery	173	2.9 (2.5–3.2)	20.2	30.1	49.7	0.08^c^	Poor	0.27^b^	Mild
Trauma	24	2.8 (1.8–3.8)	25.0	41.7	33.3	0.19^d^	—	0.25^d^	—
Diagnostic procedures	36	1.8 (0.8–2.7)	13.9	50.0	36.1	0.13^d^	—	0.47^b^	Moderate
Cancer	39	3.3 (2.4–4.1)	28.2	25.6	46.2	0.03^d^	—	0.12^d^	—
Other	167	2.4 (2.1–2.7)	41.9	37.7	20.4	0.19^a^	Mild	0.52^a^	Moderate
Unknown	22	2.5 (1.6–3.5)	40.9	31.8	27.3	0.09^d^	—	0.41^d^	—
Analgesics in the past 24 h									
No	676	1.8 (1.6–2.0)	20.3	55.0	24.7	0.18^a^	Mild	0.45^a^	Moderate
Yes	179	2.9 (2.5–3.2)	24.0	30.2	45.8	0.10^b^	Mild	0.39^a^	Moderate
Analgesic administration regimen									
* ATC*	64	2.5 (1.9–3.1)	39.1	39.1	21.9	0.19^b^	Mild	0.42^a^	Moderate
* PRN*	95	3.1 (2.7–3.6)	27.3	23.2	49.5	0.02^d^	—	0.33^b^	Mild
* ATC + PRN*	14	2.4 (1.1–3.7)	14.3	35.7	50.0	0.11^d^	—	0.57^c^	Moderate
NSAIDs									
Yes	103	2.7 (2.3–3.2)	23.3	30.1	46.6	0.11^b^	Mild	0.47^a^	Moderate
NSAIDs administration regimen									
* ATC*	25	2.2 (1.4–3.0)	20.0	40.0	40.0	0.23^c^	Mild	0.68^a^	Moderate
* PRN*	68	3.0 (2.4–3.6)	23.5	25.0	51.5	0.06^d^	—	0.37^b^	Mild
* ATC* + *PRN*	8	2.0 (0.0–4.0)	25.0	37.5	37.5	0.13^d^	—	0.44^d^	—
Opioids									
Yes	63	3.4 (2.7–4.0)	23.8	27.0	49.2	0.04^d^	—	0.17^d^	—
Opioids administration regimen									
* ATC*	30	3.2 (2.2–4.2)	20.0	33.3	46.7	0.10^d^	—	0.06^d^	—
* PRN*	24	3.5 (2.6–4.5)	37.5	16.7	45.8	−0.10^d^	—	0.20^d^	—
* ATC* + *PRN*	5	2.8 (0.1–5.5)	60.0	40.0	0.0	0.17^d^	—	0.90^d^	—
NSAIDs + opioids									
Yes	13	1.9 (0.8–3.1)	30.8	46.2	23.1	0.28^c^	Mild	0.75^b^	Strong
NSAIDs + opioids administration regimen									
* ATC*	3	0.7 (0.0–2.1)	33.3	66.8	11.1	0.50^d^	Moderate	0.88^d^	—
* PRN*	2	2.5 ( )^e^	0.0	50.0	50.0	0.00^e^	—	0.50^d^	—
* ATC* + *PRN*	8	2.6 (0.7–3.8)	37.5	37.5	25.0	0.17^d^	—	0.79^c^	Strong

For each congruence-moderator subset the table reports (1) the absolute NEP-PSRP NRS score differences as the mean and (95% confidence intervals, lower and upper levels); (2) proportions of PSRP-NEP pain categories agreement and disagreement (under- and overestimation); and (3) the results of the *κ*-statistic and the Spearman correlation coefficient analyses with both absolute numerical and category values.

CI: confidence intervals; NSAIDs: nonsteroidal anti-inflammatory drugs; *ATC*: around the clock; and *PRN*: on demand.

^a^ *P* < 0.001, ^b^ *P* < 0.01, ^c^ *P* < 0.05, ^d^statistically insignificant, ^e^insufficient cases for analysis, and ^f^assessor's assertion.

**Table 2:** Distribution and proportions of NEP and PSRP pain categories in the sample.

Moderators^a^	*n*	Pain categories, *n* (%)							
		No-pain		Mild		Moderate		Severe	
		PSRP	NEP	PSRP	NEP	PSRP	NEP	PSRP	NEP
Pain experience in the past 24 h									
No	401	301 (75.1)	336 (83.8)	39 (9.7)	43 (10.7)	39 (9.7)	16 (4.0)	22 (5.5)	6 (1.5)
Yes	461	234 (40.8)	98 (21.3)	65 (14.1)	156 (33.8)	90 (19.5)	138 (29.9)	72 (15.6)	69 (15.0)
Cause of pain									
Surgery	173	88 (50.9)	34 (19.7)	35 (20.2)	64 (37.0)	31 (17.9)	50 (28.9)	19 (11.0)	25 (14.4)
Trauma	24	6 (25.0)	3 (12.5)	2 (8.3)	5 (20.8)	9 (37.5)	10 (41.7)	7 (29.2)	6 (25.0)
Diagnostic procedures	36	27 (75.0)	19 (52.8)	1 (2.8)	11 (30.6)	4 (11.1)	4 (11.1)	4 (11.1)	2 (5.6)
Cancer	39	16 (41.0)	3 (7.7)	5 (12.8)	11 (28.2)	11 (28.2)	18 (46.2)	7 (17.9)	7 (17.9)
Other	167	84 (50.3)	33 (19.8)	21 (12.6)	57 (34.1)	30 (18.0)	50 (29.9)	32 (19.2)	27 (16.2)
Unknown	22	13 (59.1)	6 (27.3)	1 (4.5)	8 (36.4)	5 (22.7)	6 (27.3)	3 (13.6)	2 (9.1)
Analgesics in the past 24 h									
No	676	458 (67.8)	404 (59.8)	75 (11.1)	150 (22.2)	89 (13.2)	91 (13.5)	54 (8.0)	31 (4.6)
Yes	179	73 (40.8)	26 (14.5)	28 (15.6)	47 (26.3)	39 (21.8)	63 (35.2)	39 (21.8)	43 (24.0)
Analgesic administration regimen									
* ATC*	64	22 (34.4)	8 (12.5)	11 (17.2)	14 (21.9)	18 (28.1)	26 (40.6)	13 (20.3)	16 (25.0)
* PRN*	95	44 (46.3)	15 (15.8)	13 (13.7)	31 (32.6)	18 (18.9)	32 (33.7)	20 (21.1)	17 (17.9)
* ATC + PRN*	14	5 (35.7)	2 (14.3)	2 (14.3)	1 (7.1)	2 (14.3)	4 (28.6)	5 (35.7)	7 (50.0)
NSAIDs									
Yes	103	47 (45.6)	14 (13.6)	14 (13.6)	32 (31.1)	18 (17.5)	38 (36.9)	24 (23.3)	19 (18.4)
NSAIDs administration regimen									
* ATC*	25	9 (36.0)	2 (8.0)	5 (20.0)	9 (36.0)	5 (20.0)	10 (40.0)	6 (24.0)	4 (16.0)
* PRN*	68	34 (50.0)	9 (13.2)	8 (11.8)	23 (33.8)	12 (17.6)	24 (35.3)	14 (20.6)	12 (17.6)
* ATC + PRN*	8	3 (37.5)	2 (25.0)	1 (12.5)	0 (0.0)	1 (12.5)	3 (37.5)	3 (37.5)	3 (37.5)
Opioids									
Yes	63	21 (33.3)	9 (14.3)	14 (22.2)	12 (19.0)	17 (27.0)	21 (33.3)	11 (17.5)	21 (33.3)
Opioids administration regimen									
* ATC*	30	10 (33.3)	4 (13.3)	6 (20.0)	2 (6.7)	10 (33.3)	14 (46.7)	4 (13.3)	10 (33.3)
* PRN*	24	8 (33.3)	5 (20.8)	5 (20.8)	8 (33.3)	6 (25.0)	6 (25.0)	5 (20.8)	5 (20.8)
* ATC + PRN*	5	2 (40.0)	0 (0.0)	1 (20.0)	1 (20.0)	0 (0.0)	1 (20.0)	2 (40.0)	3 (60.0)
NSAIDs + opioids									
Yes	13	5 (38.5)	3 (23.1)	0 (0.0)	3 (23.1)	4 (30.8)	4 (30.8)	4 (30.8)	3 (23.1)
NSAIDs + opioids administration regimen									
* ATC*	9	3 (33.3)	2 (22.2)	0 (0.0)	3 (33.3)	3 (33.3)	2 (22.2)	3 (33.3)	2 (22.2)
* PRN*	3	2 (66.7)	1 (33.3)	0 (0.0)	0 (0.0)	0 (0.0)	2 (66.7)	1 (33.3)	0 (0.0)
* ATC + PRN*	1	0 (0.0)	0 (0.0)	0 (0.0)	0 (0.0)	1 (100.0)	0 (0.0)	0 (0.0)	1 (100.0)

Distribution and proportions of NEP and PSRP pain categories in the sample split by congruence-moderator subsets.

NEP: nurse estimation of pain; PSRP: patient self-reported pain; NSAIDs: nonsteroidal anti-inflammatory drugs; *ATC*: around the clock; and *PRN*: on demand.

^a^Assessor's assertion.

**Table 3:** Congruence categories dependence upon the PSRP category split by congruence moderators.

Moderator	*χ* ^2^	PSRP category	
		No-pain	Severe
Pain in the past 24 h			
No	330.004^a^	Cong.	
Yes	236.296^a^		Under
Cause of pain			
Surgery	96.896^a^		Under
Trauma	13.606^c^		Under
Diagnostic procedures	24.105^a^		Under
Cancer	36.897^a^		Under
Other	78.644^a^	Over	
Unknown	17.079^b^		Under
Analgesics in the past 24 h			
No	386.394^a^		Under
Yes	85.541^a^		Under
NSAIDs			
Yes	85.541^a^		Under
NSAIDs administration modality			
* ATC*	17.222^b^		Under
* PRN*	35.952^a^		Under
* ATC + PRN*	8.000^d^		—
Opioids			
Yes	32.739^a^		Under
Opioids administration modality			
* ATC*	23.476^a^		Under
* PRN*	14.267^c^		Under
* ATC + PRN*	^e^		
NSAIDs + opioids			
Yes	4.658^d^		
NSAIDs + opioids administration modality			
* ATC*	4.400^d^		
* PRN*	^e^		
* ATC + PRN*	^e^		

*χ* ^2^ analysis of the congruence categories dependence upon the PSRP category split by moderator subset (*mild* and *moderate* pain categories are not reported as there were no associations with these categories); the CCs responsible for a dependence, as found on post hoc analysis, are also reported.

In all analyses Degree of Freedom (DF) was 6 (except for pain in the past 24 h: DF = 4).

^a^ *P* < 0.001; ^b^ *P* < 0.01; ^c^ *P* < 0.05; ^d^not significant; and ^e^insufficient data for analysis.

NSAIDs: nonsteroidal anti-inflammatory drugs; *ATC*: around the clock; *PRN*: on demand; Cong.: congruence; under: underestimation; and over: overestimation.

## Additional Points

Caregivers are often inaccurate in their pain assessments and tend to misjudge pain especially in the case of symptom uncertainty. This may be driven by automated heuristics and leads to inappropriate treatment. Pain ratings agreement between inpatients and nurses could uncover bias moderators in pain assessment. Analysis of pain ratings agreement showed that medical evidence moderators such as nurses' awareness of patients' pain experience and treatment in the past 24 h bias pain evaluation agreement. Understanding the negative influence of automated heuristics driven by medical evidence moderators on pain evaluation and avoiding them may render pain assessment more accurate.

## References

[1] Gunnarsdottir S., Donovan H. S., Ward S. (2003). Interventions to overcome clinician- and patient-related barriers to pain management. *Nursing Clinics of North America*.

[2] Solomon P. (2001). Congruence between health professionals' and patients' pain ratings: a review of the literature. *Scandinavian Journal of Caring Sciences*.

[3] Melotti R. M., Samolsky Dekel B. G., Carosi F. (2009). Categories of congruence between inpatient self-reported pain and nurses evaluation. *European Journal of Pain*.

[4] Kappesser J., Williams A. C. D. C. (2010). Pain estimation: asking the right questions. *Pain*.

[5] Prkachin K. M., Solomon P. E., Ross J. (2007). Underestimation of pain by health-care providers: towards a model of the process of inferring pain in others. *Canadian Journal of Nursing Research*.

[6] Puntillo K., Neighbor M., O'Neil N., Nixon R. (2003). Accuracy of emergency nurses in assessment of patient's pain. *Pain Management Nursing*.

[7] Kappesser J., Williams A. C. D. C., Prkachin K. M. (2006). Testing two accounts of pain underestimation. *Pain*.

[8] Prkachin K. M., Solomon P., Hwang T., Mercer S. R. (2001). Does experience influence judgments of pain behaviour? Evidence from relatives of pain patients and therapists. *Pain Research and Management*.

[9] Prkachin K. M., Mass H., Mercer S. R. (2004). Effects of exposure on perception of pain expression. *Pain*.

[10] Prkachin K. M., Rocha E. M. (2010). High levels of vicarious exposure bias pain judgments. *Journal of Pain*.

[11] Rash J. A., Prkachin K. M., Campbell T. S. (2015). Observer trait anxiety is associated with response bias to patient facial pain expression independent of pain catastrophizing. *Pain Research and Management*.

[12] Tait R. C., Chibnall J. T. (1997). Physician judgments of chronic pain patients. *Social Science and Medicine*.

[13] Van der Does A. J. W. (1989). Patients' and nurses' ratings of pain and anxiety during burn wound care. *Pain*.

[14] Melotti R. M., Samolsky-Dekel B. G., Ricchi E. (2005). Pain prevalence and predictors among inpatients in a major Italian teaching hospital. A baseline survey towards a pain free hospital. *European Journal of Pain*.

[15] Marquié L., Raufaste E., Lauque D., Mariné C., Ecoiffier M., Sorum P. (2003). Pain rating by patients and physicians: evidence of systematic pain miscalibration. *Pain*.

[16] Tait R. C., Chibnall J. T. (1994). Observer perceptions of chronic low back pain. *Journal of Applied Social Psychology*.

[17] Tait R. C., Chibnall J. T., Kalauokalani D. (2009). Provider judgments of patients in pain: seeking symptom certainty. *Pain Medicine*.

[18] Iafrati N. S. (1986). Pain on the burn unit: patient vs nurse perceptions. *Journal of Burn Care and Rehabilitation*.

[19] Myles P. S., Gin T., Myles P. S., Gin T. (2000). Regression and correlation. *Statistical Methods for Anaesthesia and Intensive Care*.

[20] Collins S. L., Moore R. A., McQuay H. J. (1997). The visual analogue pain intensity scale: what is moderate pain in millimetres?. *Pain*.

[21] Bartfield J. M., Salluzzo R. F., Raccio-Robak N., Funk D. L., Verdile V. P. (1997). Physician and patient factors influencing the treatment of low back pain. *Pain*.

[22] Chibnall J. T., Tait R. C. (1995). Observer perceptions of low back pain: effects of pain report and other contextual factors. *Journal of Applied Social Psychology*.

[23] Chibnall J. T., Tait R. C., Ross L. R. (1997). The effects of medical evidence and pain intensity on medical student judgments of chronic pain patients. *Journal of Behavioral Medicine*.

[24] Chibnall J. T., Tait R. C., Merys S. C. (2000). Disability management of low back injuries by employer-retained physicians: ratings and costs. *American Journal of Industrial Medicine*.

[25] Chibnall J. T., Dabney A., Tait R. C. (2000). Internist judgments of chronic low back pain. *Pain Medicine*.

[26] Igier V., Mullet E., Sorum P. C. (2007). How nursing personnel judge patients' pain. *European Journal of Pain*.

[27] Marquié L., Sorum P. C., Mullet E. (2007). Emergency physicians' pain judgments: cluster analyses on scenarios of acute abdominal pain. *Quality of Life Research*.

[28] Tait R. C., Chibnall J. T. (2002). Pain in older subacute care patients: associations with clinical status and treatment. *Pain Medicine*.

[29] Todd K. H., Lee T., Hoffman J. R. (1994). The effect of ethnicity on physician estimates of pain severity in patients with isolated extremity trauma. *The Journal of the American Medical Association*.

[30] Robinson M. E., Wise E. A. (2003). Gender bias in the observation of experimental pain. *Pain*.

[31] Tait R. C., Chibnall J. T. (2001). Work injury management of refractory low back pain: relations with ethnicity, legal representation and diagnosis. *Pain*.

[32] Grossman S. A., Sheidler V. R., Swedeen K., Mucenski J., Piantadosi S. (1991). Correlation of patient and caregiver ratings of cancer pain. *Journal of Pain and Symptom Management*.

[33] Zalon M. L. (1993). Nurses' assessment of postoperative patients' pain. *Pain*.

[34] Gelinas C., Puntillo K. A., Joffe A. M., Barr J. (2013). A validated approach to evaluating psychometric properties of pain assessment tools for use in nonverbal critically ill adults. *Seminars in Respiratory and Critical Care Medicine*.

[35] AGS Panel on Persistent Pain in Older Persons (2002). The management of persistent pain in older persons. *Journal of the American Geriatrics Society*.

[36] Pasero C., McCaffery M. (2001). The undertreatment of pain. *American Journal of Nursing*.

[37] Cocchiarella L., Andersson G. B. J. (2001). *Guides to the Evaluation of Permanent Impairment*.

